# Cost-effectiveness Analysis in R Using a Multi-state Modeling Survival Analysis Framework: A Tutorial

**DOI:** 10.1177/0272989X16651869

**Published:** 2016-06-08

**Authors:** Claire Williams, James D. Lewsey, Andrew H. Briggs, Daniel F. Mackay

**Affiliations:** Health Economics and Health Technology Assessment, Institute of Health and Wellbeing, University of Glasgow, Glasgow, UK (CW, JDL, AHB); Public Health, Institute of Health and Wellbeing, University of Glasgow, Glasgow, UK (DFM)

**Keywords:** Markov models, cost-effectiveness analysis, probabilistic sensitivity analysis, survival analysis

## Abstract

This tutorial provides a step-by-step guide to performing cost-effectiveness analysis using a multi-state modeling approach. Alongside the tutorial, we provide easy-to-use functions in the statistics package R. We argue that this multi-state modeling approach using a package such as R has advantages over approaches where models are built in a spreadsheet package. In particular, using a syntax-based approach means there is a written record of what was done and the calculations are transparent. Reproducing the analysis is straightforward as the syntax just needs to be run again. The approach can be thought of as an alternative way to build a Markov decision-analytic model, which also has the option to use a state-arrival extended approach. In the state-arrival extended multi-state model, a covariate that represents patients’ history is included, allowing the Markov property to be tested. We illustrate the building of multi-state survival models, making predictions from the models and assessing fits. We then proceed to perform a cost-effectiveness analysis, including deterministic and probabilistic sensitivity analyses. Finally, we show how to create 2 common methods of visualizing the results—namely, cost-effectiveness planes and cost-effectiveness acceptability curves. The analysis is implemented entirely within R. It is based on adaptions to functions in the existing R package mstate to accommodate parametric multi-state modeling that facilitates extrapolation of survival curves.

Markov decision-analytic models^1–3^ are a widely used modeling approach in cost-effectiveness analysis^4^ and are typically built in spreadsheet-based packages or commercial packages such as TreeAge.^5^ Spreadsheets, especially Microsoft Excel, have the advantage of being familiar, widely available, simple to use, and easy to share with others. However, the calculations are often over several (linked) sheets rather than being contained together on one page. It can be hard to keep track of modifications to the model and the resulting output when cells are changed, especially if altered accidently. In addition, each time a parameter is changed, the previous analysis is lost, so retaining a full record of the analysis requires multiple workbooks to be kept.

This article demonstrates how a cost-effectiveness analysis can be carried out within a multi-state modeling survival analysis framework using the statistical software R,^6^ which is freely available under the GNU General Public Licence. As suggested by others,^7^ using a script-based approach with statistical software, rather than a spreadsheet-based package, has many advantages when carrying out cost-effectiveness analysis. First, calculations are transparent, with the coding script providing a written record of what was done. Second, it is simple to reproduce the analysis—the lines of code just need to be run again. Third, potential errors are easier to spot as code can be interactively debugged line by line to identify the problem. Last, flexible graphics facilities are available within R. Cost-effectiveness analysis using multi-state modeling in R has been introduced elsewhere.^8^ This article builds on this and illustrates how the Markov property can be empirically tested by using a “state-arrival extended” multi-state model. A state-arrival extended multi-state model includes a covariate representing patients’ histories such as time in the previous state. The significance, statistically and clinically, of the covariate can help in deciding whether the Markov assumption is reasonable and therefore the approach to take for the analysis.

The aims of this tutorial article are 1) to introduce the “state-arrival extended” multi-state model as a tool to test the Markov property and 2) to provide a step-by-step guide to how multi-state modeling can be used for carrying out a cost-effectiveness analysis, including discounting of costs/benefits and deterministic and probabilistic sensitivity analyses. The R code is written in the form of functions so that those unfamiliar with R code can still use them. All that needs to be changed are the customizable arguments given to the functions, such as the number of transitions and covariate information, the discount rate, and the time horizon. The functions are based on adaptions to the existing R package mstate.^9^ It is assumed that individual patient data are available from the trial/study and that the times of transitions are known exactly. It is still possible to use the approach when individual patient data are not available; this scenario will be briefly considered in the Discussion.

## Decision-Analytic Modeling Expressed in the Multi-state Model Survival Analysis Framework

Multi-state modeling builds survival regression models for each of the transitions. Survival times are treated as continuous variables, rather than being measured in discrete cycles as is usually the case in decision-analytic modeling. Therefore, the arrows normally seen in a model diagram that leave and reenter a state—to indicate patients who remain in that state for the length of a cycle—are not applicable.

Two ways to treat time in multi-state modeling are the clock-forward and clock-reset approach.^10^ With the clock-forward approach, time is measured from the initial state, whereas with the clock-reset approach, every time a patient reaches a new state, the clock is set back to zero, thereby only measuring time in the current (new) state. The clock-forward approach is a Markov model because the property that movement from the present state does not depend on history is inherent. The timescale of the clock-reset approach does depend on history, and models fitted using this approach are referred to as semi-Markov rather than Markov.^10^

One way of testing whether the Markov property is violated is to include in the model a covariate representing history. Such models have been termed *state-arrival extended* multi-state models. They are described in Putter and others^10^ as a model of an “i → j transition hazard that depends on the time of arrival at state i.” Inclusion of a covariate for the time in the previous state, or any function thereof, could therefore aid the decision of whether the Markov assumption is reasonable. We use a Markov state-arrival extended model to help inform this decision. We then proceed to use a semi-Markov approach for all our modeling because the Markov property is not thought to hold.

**Figure 1:**
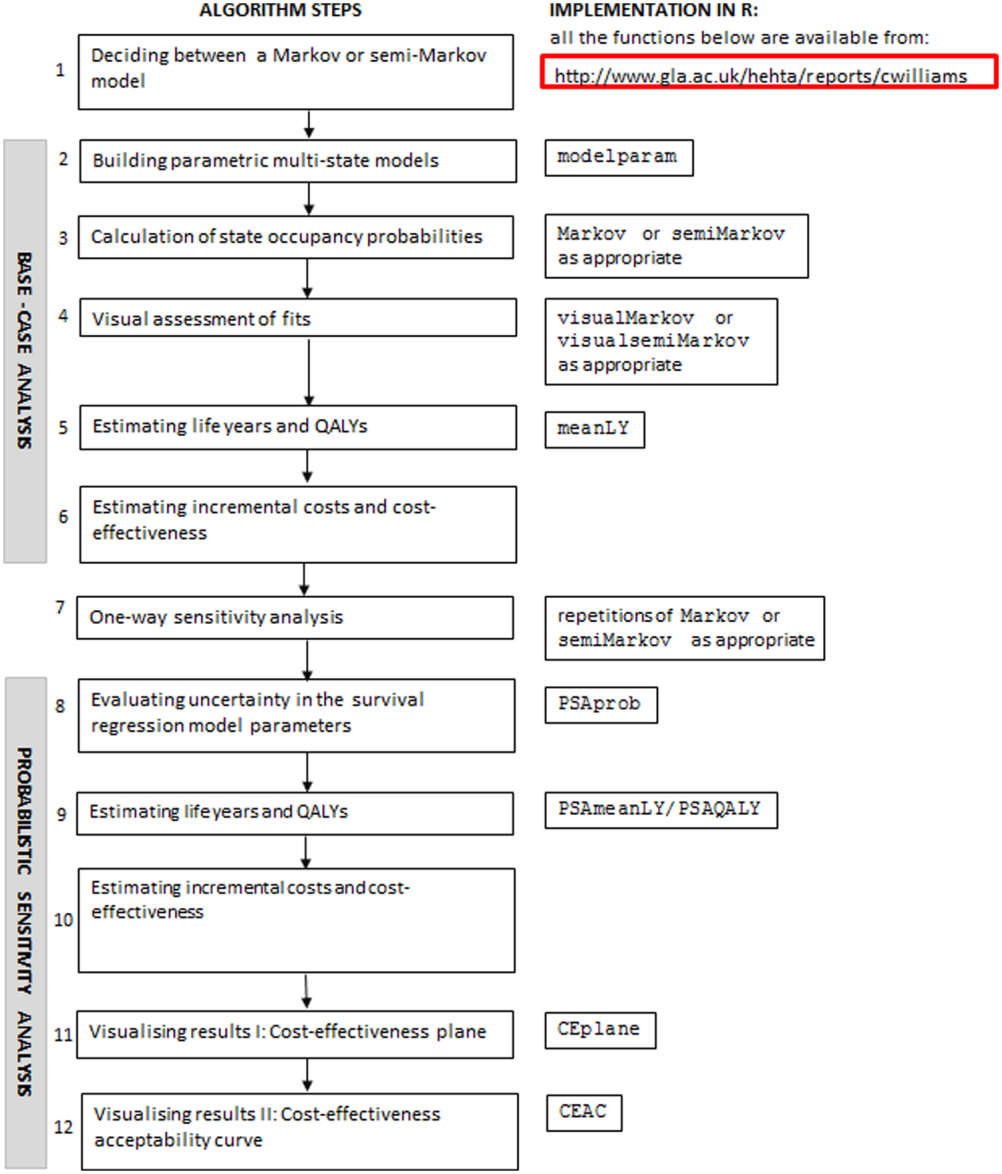
Algorithm for health economic modeling using a multi-state modeling framework.

Figure 1 shows an algorithm that can be used to perform health economic modeling in a multi-state modeling survival analysis framework. All of the functions included in Figure 1 are adaptions written by the authors to the functions already available in the mstate package in R. They are all available from www.gla.ac.uk/hehta/reports/cwilliams. In the steps detailed below, any words in Courier New font, other than mstate, probtrans, or mssample, refer to one of the adapted functions.

An explanation of each of the steps is given as follows.

### Step 1: Deciding Whether to Accept the Markov Property

Building a Cox Markov state-arrival extended multi-state model can aid the decision of whether the Markov assumption is reasonable or not. Such models are Cox in the usual semi-parametric sense that the baseline hazard does not follow a specified distribution, and Markov in the sense that time is measured from the initial state for every transition. The state-arrival extended aspect of the modeling of a particular transition could, for example, include a covariate for time spent in the immediate previous state. A statistically significant covariate effect would then provide evidence that the Markov property does not hold. However, if the effect is small such that it is not of practical importance, then the analysis could safely proceed as if the Markov property did hold.

### Step 2: Building Parametric Multi-state Models

Many models contemplate death as an absorbing state (i.e., a state from which a transition to another state is not possible). If the period of follow-up of the study is such that for every patient, his or her whole lifetime since entry into the study is represented, then the functions in mstate, which are based on Cox semi-parametric proportional hazards models, can be used without further modification. Similarly, where there is an absorbing state other than death observed for every patient, the functions in mstate remain appropriate. From now on, it is thus assumed, with no loss of generality, that death is the sole absorbing state in the models.

Often death is not observed for every patient due to limitations of follow-up. When this is the case and the analysis has a lifetime horizon, extrapolation of survival is required, as recommended by the UK National Institute for Health and Care Excellence (NICE) Decision Support Unit.^11^ This is necessary because the cost-effectiveness calculations need an estimate of mean survival. A popular choice to achieve the necessary extrapolation is to assume a parametric distribution when modeling the hazards. The modelparam function allows the user to fit either a Markov or semi-Markov model to a transition with a choice of several standard parametric distributions: notably the exponential, Weibull, Gompertz, lognormal, log-logistic, and generalized gamma. This function, as well as the Markov and semiMarkov function introduced in the next step, also accommodates state-arrival extended models due to the customizable covariate arguments.

### Step 3: Calculating State Occupancy Probabilities

The Markov and semiMarkov functions adapt the functionality already available in the mstate package in R to accommodate hazards from a range of different distributions. They both build models that assume the desired distribution for the hazards, similar to step 2, although now models for every transition are included. The cumulative hazards for each transition are then combined in such a way that they can then be used by the appropriate functions in mstate to calculate state occupancy probabilities. The Markov function uses the probtrans function in mstate to calculate probabilities encoding exact Markov prediction formulas, similar to the Markov traces used in spreadsheet-based approaches. The semiMarkov function instead calculates probabilities using the mssample function, which simulates all relevant paths (all possible transition journeys) through the multi-state model.^12^ The functions have several customizable arguments, such as distribution for each transition, the number of transitions, number of covariates, values of covariates evaluated in each transition, and time horizon.

### Step 4: Visual Assessment of Fits

Visual assessments of fits can help in choosing the distribution to use for each transition. A balance between a good fit to the observed data and the necessary extrapolation to the time horizon is desirable. This can be assessed by plotting the observed proportion in a given state alongside the predicted probability of being in that state from the model(s) over the observed period of the trial and then again extended to the target time horizon.

The visualMarkov and visualsemiMarkov functions plot, for Markov and semi-Markov models, respectively, the predicted probability of being in a given state over time using different distributions alongside the observed proportion in that state over time. The functions can also accommodate comparisons of observed proportions v. predictions of not reaching an absorbing state (i.e., “1 – probability” of reaching an absorbing state). Other forms of assessment of model fit such as the Akaike information criterion (AIC)^13^ are popular. However, they only provide information about the fit to the observed data, while often discounting for some measure of model complexity and not on how reasonable the extrapolation looks. When assessing model fit, it is advisable to consider several different aspects, allowing the observed fit and extrapolation to be jointly assessed. When faced with a competing risks scenario while modeling transition hazards, however, such AIC calculations are not appropriate and therefore visual assessment can be the only option.

### Step 5: Estimating Life Years and Quality-Adjusted Life Years

The mean life years in a particular state for a particular treatment can be calculated using the meanLY function. The mean life years gained from a treatment for that particular state can then be obtained by calculating the difference in mean life years between the 2 treatments. When each state has a fixed utility, the mean quality-adjusted life years (QALYs) can easily be derived by multiplying the mean life years by the utility weight.

### Step 6: Estimating Incremental Costs and Cost-Effectiveness

An R function is not provided for this step as it is very data dependent. However, the R commands for the illustration later in the article are available from www.gla.ac.uk/hehta/reports/cwilliams.

### Step 7: Structural/One-Way Sensitivity Analysis

One-way sensitivity analysis can be achieved by repeated analyses, modifying arguments in the Markov and/or semiMarkov functions each time or by varying the costs. However, caution is advised when using such an approach because parameters are only changed one at a time in isolation.^14^

### Step 8: Uncertainty in the Survival Regression Model Parameters

The function PSAprob produces state occupancy probabilities for each draw. It starts by building the base-case model for each transition. The correlation between the parameters in the model is then taken into account by using the Cholesky decomposition to provide correlated draws from a multivariate normal distribution.^15^ The vector of correlated variables contains draws of the relevant parameters of the model used to create cumulative hazards underpinning state occupancy probabilities.

### Step 9: Estimating Life Years and QALYs

The PSAmeanLY function calculates the mean life years in a particular state for a particular treatment for each draw and then computes the mean across sampled draws. The PSAQALY function calculates the mean QALYs in a particular state for a particular treatment for each draw. The overall mean can be then obtained by taking the mean of the result.

### Step 10: Estimating Incremental Costs and Cost-Effectiveness

Cost parameters are usually also considered in probabilistic sensitivity analyses.^4^ While no R function is provided here due to this step being very data dependent, the R commands for the illustration later in the article available from http://www.gla.ac.uk/hehta/reports/cwilliams include this step.

### Step 11: Visualizing Results I: Cost-Effectiveness Plane

The incremental (discounted) QALY needed for a plot of the cost-effectiveness plane^4^ can be obtained using repeated evaluations of the PSAQALY function. To obtain the incremental QALY, first the QALY for each treatment group should be calculated. This can be achieved by using the PSAQALY function for each relevant state/treatment group combination. The QALYs for each relevant state for the treatment of interest should then be added together, with the corresponding information for the control treatment subtracted from the result. The CEplane function can then be used to plot a cost-effectiveness plane.

### Step 12: Visualizing Results II: Cost-Effectiveness Acceptability Curve

The total (discounted) QALY for each treatment arm needed for a plot of the cost-effectiveness acceptability curve^16^ can be obtained using repeated evaluations of the PSAQALY function. PSAQALY can be applied for each relevant state/treatment combination with the evaluations for each treatment group then added together. The CEAC function can then be used to plot a cost-effectiveness acceptability curve.

## Illustrative Example

### Data Set Used for Illustration

The data used in this article are based on a trial comparing rituximab in combination with fludarabine and cyclophosphamide (RFC) v. fludarabine and cyclophosphamide alone (FC) for the first-line treatment of chronic lymphocytic leukemia (CLL-8).^17^ It was the main source of data used by the manufacturer in their submission to NICE in the United Kingdom for the specific technology appraisal TA174.^18,19^ The trial reported the outcomes of progression-free survival and overall survival for each patient, allowing focus to be on 3 states (progression-free, progression, and death) and the transitions between them. There were 403 patients in the RFC arm and 407 patients in the FC arm. There were 106 progressions, 23 deaths after progression, and 21 deaths without progression among those in the RFC arm. In the FC arm, there were 148 progressions, 27 deaths after progression, and 26 deaths without progression. Patients were in the trial for up to 4 years, and not all were observed to the end of their lives. This meant extrapolation of survival was necessary to obtain a representation of the whole duration of life since entry into the trial. It was estimated that only 1.3% of the cohort would survive beyond 15 years^19(p109)^ and therefore a time horizon of 15 years was used. The published Kaplan-Meier curves in the manufacturer’s report^19^ were digitized using Enguage^20^ to generate the data for the analysis.

### Transitions Modeled and Initial Modeling

**Figure 2:**
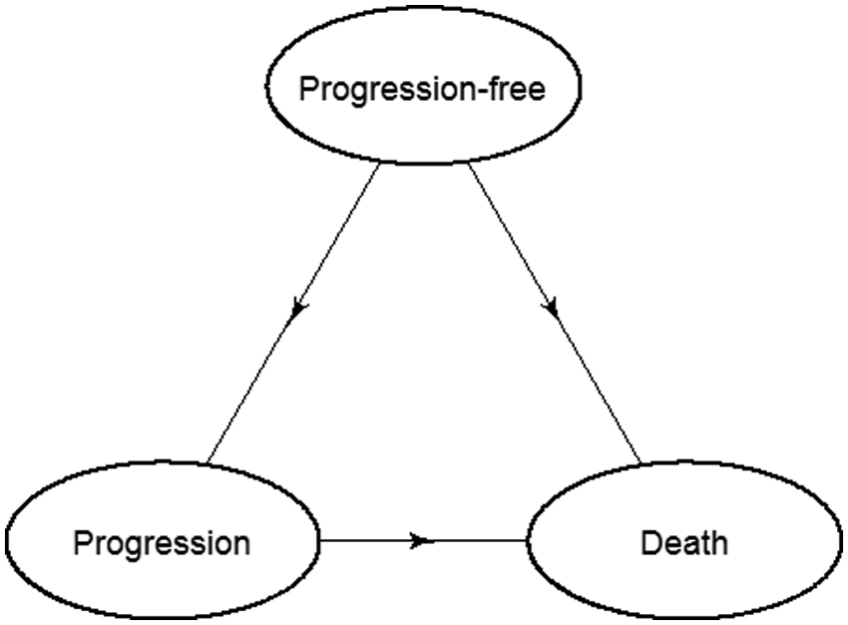
Transition diagram for multi-state model showing the 3 transitions: 1) progression-free to progression, 2) progression-free to death, and 3) progression to death.

The state transition model is illustrated in Figure 2.

The Web page http://www.gla.ac.uk/hehta/reports/cwilliams includes the syntax with the R commands used for all the analysis in this example.

**Figure 3:**
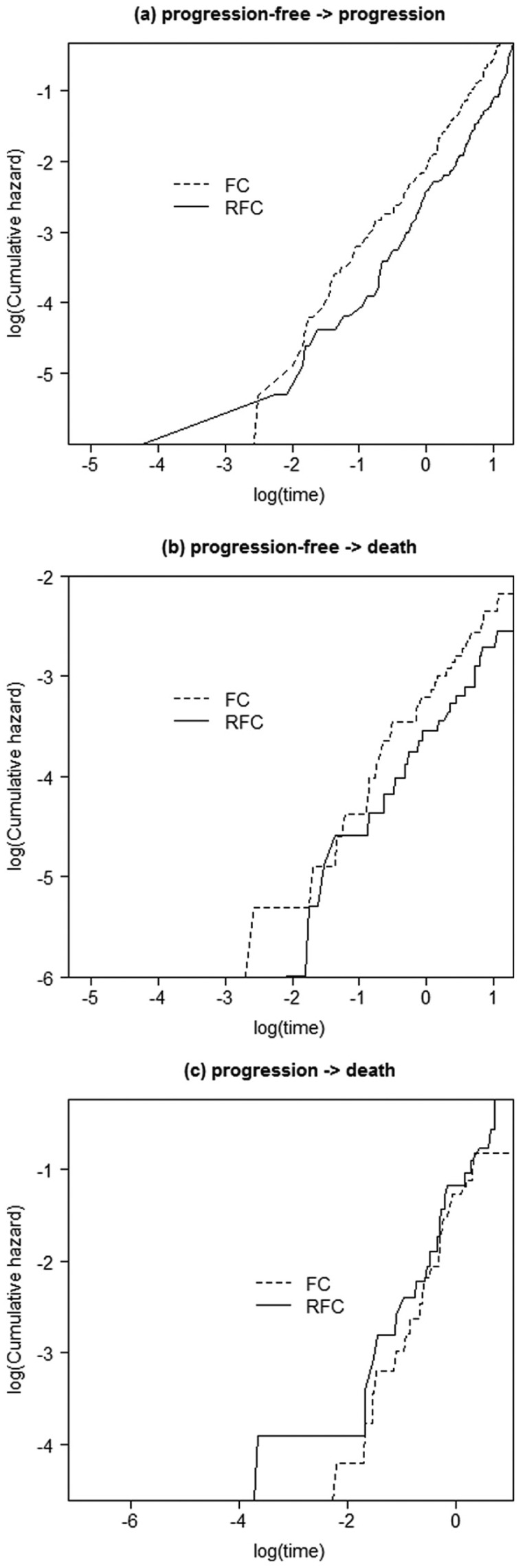
Log-log plots for each transition. FC, fludarabine and cyclophosphamide; RFC, rituximab, fludarabine, and cyclophosphamide.

The analysis began by considering whether the proportional hazards (PH) assumption was reasonable for each transition, to determine whether it was appropriate to consider PH models. Figure 3 shows a log-log plot for treatment, the only covariate under consideration, for each of the respective transitions.

It can be seen in each of the plots (Figure 3a–c) that the lines were reasonably parallel, with any crossing of the lines due to the lack of a treatment effect rather than any major violation of the PH assumption.

**Figure 4:**
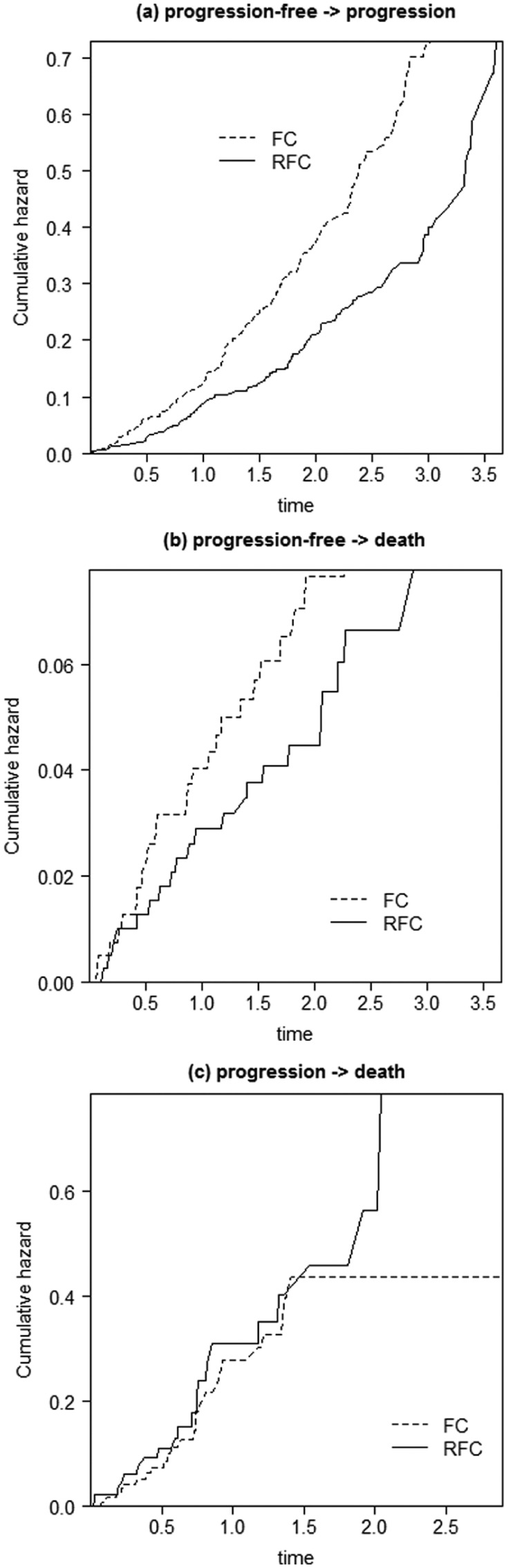
Cumulative hazard plots for each transition. FC, fludarabine and cyclophosphamide; RFC, rituximab, fludarabine, and cyclophosphamide.

Figure 4 shows a cumulative hazard v. time plot for treatment in each of the respective transitions.

The progression → death plot (Figure 4c) reflected the lack of a treatment effect. The other plots (Figure 4a,b) showed lines that diverged, indicating considering a distribution that facilitates increasing hazards would be appropriate.

Since there was no suggestion of a severe violation of the PH assumption from Figure 3 or 4, proportional hazards models were considered for the analysis.

To help decide whether the Markov property held, a Cox Markov state-arrival extended model for progression → death was initially fitted. Table 1 shows the results of fitting this model.

**Table 1:** Results of a Cox Markov State-Arrival Extended Model

	Hazard Ratio (95% CI)	*P* Value
Treatment	1.555 (0.874, 2.766)	0.133
Time spent progression-free	0.413 (0.215, 0.794)	0.008

The time spent progression-free was found to have a statistically significant association with death after progression (*P* = 0.008). The hazard ratio point estimate and 95% confidence interval were below 1, indicating that the longer the time spent progression-free, the lower the risk of death after reaching the progression state. For each increase of 1 year in the progression-free state, the hazard of death reduced by 58.7%. The point estimate of 58.7% equates to, for those in the FC treatment arm who spent 1 year in the progression-free state, an absolute risk of death of 72.9% at 4 years after reaching the progression state. The corresponding figure for those in the FC arm who spent 2 years in the progression-free state was 41.7%. The equivalent figures for the RFC arm were 86.9% and 56.8%. The effect of time in the previous state was of a size likely to be of practical importance, both in relative and absolute terms. Therefore, there was evidence to suggest the Markov property did not hold, indicating that a semi-Markov model would be more appropriate on this occasion.

### Base-Case Analysis

Parametric semi-Markov models were then fitted to allow both a relaxation of the Markov assumption and extrapolation of survival. The choice of distribution for each transition was considered. The progression → death transition was considered first because it did not involve a competing risk scenario.

**Figure 5:**
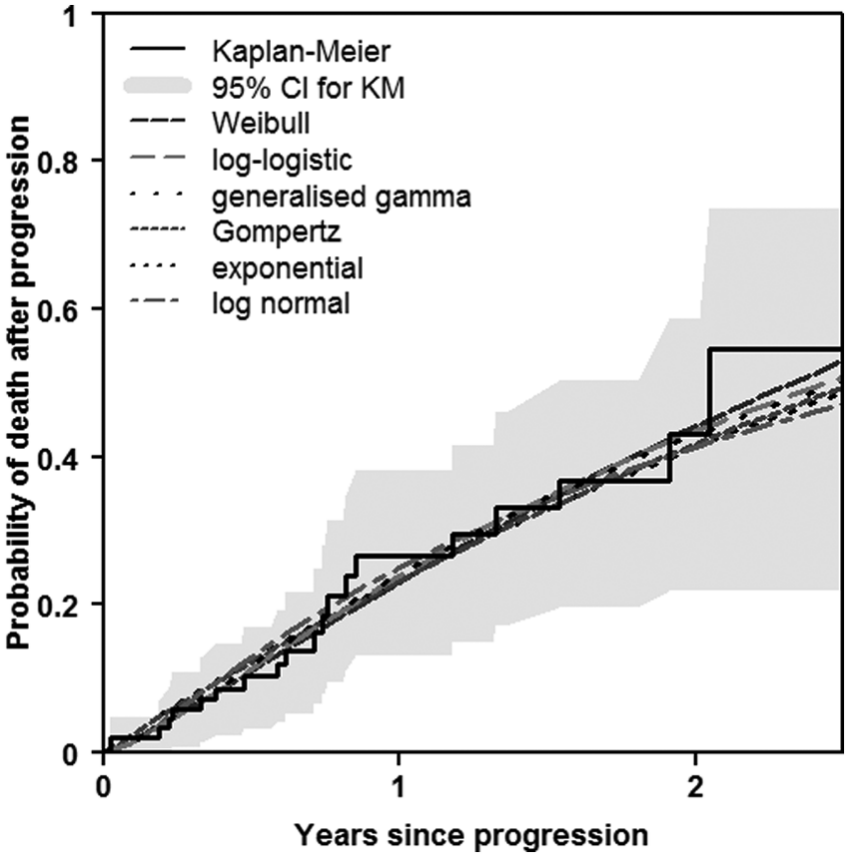
Observed and predicted proportions for progression → death: trial observation period (RFC treatment only). KM, Kaplan-Meier; RFC, rituximab, fludarabine, and cyclophosphamide.

Figure 5 shows, over the trial observation period, the observed and predicted proportion of deaths after progression using 6 different candidate distributions. For brevity, only the RFC treatment arm is shown. It can be seen that the predictions were reasonably similar. The lowest AIC value was seen for the log-logistic distribution, suggesting that it provided the best fit of the candidate distributions, although there was little to choose between the distributions (Table 2).

**Table 2:** Akaike Information Criterion (AIC) Statistics from Modeling Progression → Death

Distribution	AIC
Exponential	224.3
Log-logistic	223.5
Weibull	224.9
Lognormal	224.7
Gompertz	226.3
Generalized gamma	225.8

**Figure 6:**
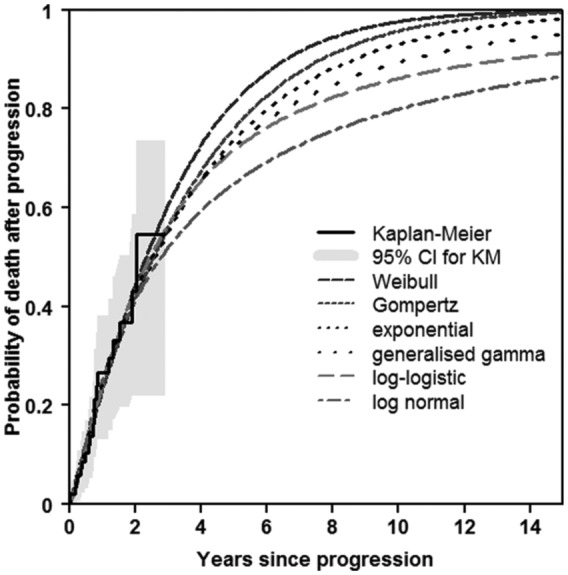
Observed and predicted proportions for progression → death: extrapolation to 15 years (RFC treatment only). KM, Kaplan-Meier; RFC, rituximab, fludarabine, and cyclophosphamide.

The time horizon of the model was 15 years. Therefore, in addition to comparing the fit over the period of the observed data, interest was also in assessing how the survival estimates extend out to 15 years. Figure 6 shows observed v. predicted probabilities over 15 years. The Weibull, Gompertz, and exponential distributions all appeared to represent a time horizon of 15 years in the sense that the probability of death was close to 1 by 15 years.

After inspecting the plots for the other group (not shown), a Gompertz distribution appeared to be a reasonable distribution to use for this transition. Consideration of the distributions to use for the progression-free → progression and progression-free → death transitions is contained in Appendix 1 as online supplementary material. These 2 transitions involved competing risks, and because AIC statistics are not appropriate when modeling transition hazards in a competing risks scenario, the choice of distributions to use was based on visual assessment of the plots alone. The Gompertz and generalized gamma distributions appeared to provide a reasonable fit for the progression-free → progression and progression-free → death transitions, respectively, over the trial observation and extrapolation period.

Table 3 shows the mean costs used with the multi-state modeling approach.

**Table 3:** Assumptions Used for Mean Costs

	RFC	FC	Incremental
**Mean cost of PFS**	**£18,693**	**£6,769**	**£11,924**
Costs of rituximab^a^	£10,113	£0	£10,113
Administration costs of rituximab^b^	£1224	£0	£1224
Cost of fludarabine^a^	£2776	£2790	−£14
Administration costs of fludarabine^b^	£1109	£1115	−£6
Costs of cyclophosphamide^a^	£21	£22	£0
Administration costs of cyclophosphamide^b^	£1109	£1115	−£6
Cost of supportive care in PFS^b^	£1109	£860	£249
Cost of bone marrow transplantation^b^	£592	£360	£231
Cost of blood transfusions^c^	£640	£507	£133
**Mean cost of progression**	**£7224**	**£8740**	**−£1516**
Cost of supportive care in progression^b^	£2537	£3069	−£532
Cost of second-line and subsequent therapy^a^	£4687	£5671	−£984
**Mean total cost**	**£25,917**	**£15,508**	**£10,408**

FC, fludarabine and cyclophosphamide; PFS, progression-free survival; RFC, rituximab, fludarabine, and cyclophosphamide.

a Source: British National Formulary 56. http://www.medicinescomplete.com/mc/bnf/current/

b Source: Department of Health. NHS reference costs 2006/2007. http://webarchive.nationalarchives.gov.uk/20130107105354/http://www.dh.gov.uk/en/Publicationsandstatistics/Publications/PublicationsPolicyAndGuidance/DH_082571

c Sources: Agrawal S, Davidson N, Walker M, et al. Assessing the total costs of blood delivery to hospital oncology and haematology patients. Curr Med Res Opin. 2006;22(10):1903–9. Curtis L. Unit costs of health and social care. Personal Social Services Research Unit, Kent, United Kingdom; 2007.

Most of the mean costs were not related to the time spent in relevant health states. However, cost of supportive care while progression-free, cost of supportive care while in progression, and cost of second-line and subsequent therapy while in progression were all associated with time spent in relevant states. Therefore, the mean life years in the appropriate states from the multi-state model were used in the calculation of these costs. All other costs were taken from the original manufacturer’s submission.^19(pp127–31)^

The cost per life year or QALY gained, commonly known as the incremental cost-effectiveness ratio (ICER), was then calculated. Table 4 shows the results of the base-case analysis in terms of the mean life years in each state, the mean QALYs in each state, the mean costs, the cost per life year gained, and that per QALY gained. Utilities of 0.8 and 0.6 were assumed for progression-free and progression, respectively.^21^

**Table 4:** Base-Case Analysis Results

	RFC	FC	Incremental
**Mean life years**	**5.82**	**5.60**	**0.21**
Mean life years progression-free	3.30	2.56	0.74
Mean life years in progression	2.52	3.04	−0.53
**Mean QALYs**	**4.15**	**3.87**	**0.28**
Mean QALYs progression-free	2.64	2.05	0.59
Mean QALYs in progression	1.51	1.83	−0.32
**Mean total cost**	**£25,917**	**£15,508**	**£10,408**
Cost per life year gained			£48,772
**Cost per QALY gained**			**£37,665**

FC, fludarabine and cyclophosphamide; QALY, quality-adjusted life year; RFC, rituximab, fludarabine, and cyclophosphamide.

An increment of mean life years (QALYs) while progression-free was found of 0.74 (0.59). However, a relatively large decrement of mean life years (QALYs) of −0.53 (−0.32) was found while in progression. Therefore, the benefits overall of mean life years gained of 0.21 and mean QALYs gained of 0.28 were relatively small. This led to a cost per QALY gained of close to £38,000, in excess of the £30,000 willingness-to-pay threshold commonly used in the United Kingdom, and therefore the treatment was deemed not cost-effective.

### Structural/One-Way Sensitivity Analyses

Table 5 details the base-case assumptions and one-way sensitivity analyses. A more comprehensive sensitivity analysis specifically considering the distributions used for each transition is contained in Appendix 2 as online supplementary material.

**Table 5:** Structural/One-Way Sensitivity Analyses

Base Case Assumption	Sensitivity Analysis	Cost per QALY Gained
	***Base case***	**£37,665**
Time horizon of 15 years	Time horizon extended to 20 years	£39,946
Observed treatment effects persist to the end of the time horizon	Treatment effect no longer persists in extrapolation	£85,132
Utilities: PFS = 0.8; progressed = 0.6	Utilities: PFS = 0.9; progressed = 0.5	£25,808
Utilities: PFS = 0.8; progressed = 0.6	Utilities: PFS = 0.75; progressed = 0.65	£48,897
Oral administration of FC	IV infusion of FC = actual dose from trial	£34,632
Oral administration of FC	IV infusion of FC = recommended dose	£37,088
Adverse event costs excluded	Inclusion of adverse event costs	£37,966
	Monthly supportive care cost increase by 50%	£37,153
	Monthly supportive care cost decrease by 50%	£38,177
	Drug administration cost upper quartile	£41,903
	Drug administration cost lower quartile	£34,828

FC, fludarabine and cyclophosphamide; IV, intravenous; PFS, progression-free survival; QALY, quality-adjusted life year.

It is not always appropriate to assume that the treatment effect observed within the trial persisted beyond that period. Therefore, as part of the sensitivity analysis, we consider an alternative assumption whereby the treatment effect no longer persists in the unobserved period. It can be seen from Table 5 that there was some uncertainty with regard to the cost-effectiveness, especially if a willingness-to-pay threshold of £30,000 per QALY gained was used. When the gap between the utilities was widened, the cost per QALY gained was £25,808. In all other analyses, there was no change in the conclusion that the treatment was not cost-effective.

### Probabilistic Sensitivity Analysis

Each cost parameter involved in the manufacturer’s probabilistic sensitivity analysis was assumed to follow a Beta Pert distribution.^22^ For the purposes of this illustration, we used the same distributions. However, analysts are free to consider a range of different distributions, as appropriate, with their own studies. Table 6 shows the mean base-case estimates together with the ranges used to generate the distributions. The particular Beta Pert distribution chosen for the cost of monthly supportive care and second-line and subsequent therapy while in progression was dependent on the mean life years in progression. All other distribution parameters values were as presented by the manufacturer.^19(pp137–8)^

**Table 6:** Beta Pert Distributions Used in Probabilistic Sensitivity Analysis for Cost Parameters

Costs	Base Case	Minimum	Maximum
Monthly supportive care cost while in PFS^a^	£28	£14	£42
Monthly supportive care and second-line and subsequent therapy cost while in progression^a^	£218.65	£109.32	£327.97
Administration—deliver exclusively oral chemotherapy^a^	£280	£174	£482
Administration—deliver complex chemotherapy, including prolonged infusional treatment at first attendance^a^	£430	£210	£795
Bone marrow transplant^a^	£47,565.05	£34,318.25	£54,646.47
Blood transfusion^b^	£289.73	£173.84	£405.62
1 Unit of blood	£161.11	£96.67	£225.26

PFS, progression-free survival.

a Source: Department of Health. NHS reference costs 2006/2007. http://webarchive.nationalarchives.gov.uk/20130107105354/http://www.dh.gov.uk/en/Publicationsandstatistics/Publications/PublicationsPolicyAndGuidance/DH_082571

b Source: Agrawal S, Davidson N, Walker M, et al. Assessing the total costs of blood delivery to hospital oncology and haematology patients. Curr Med Res Opin. 2006;22(10):1903–9.

**Figure 7:**
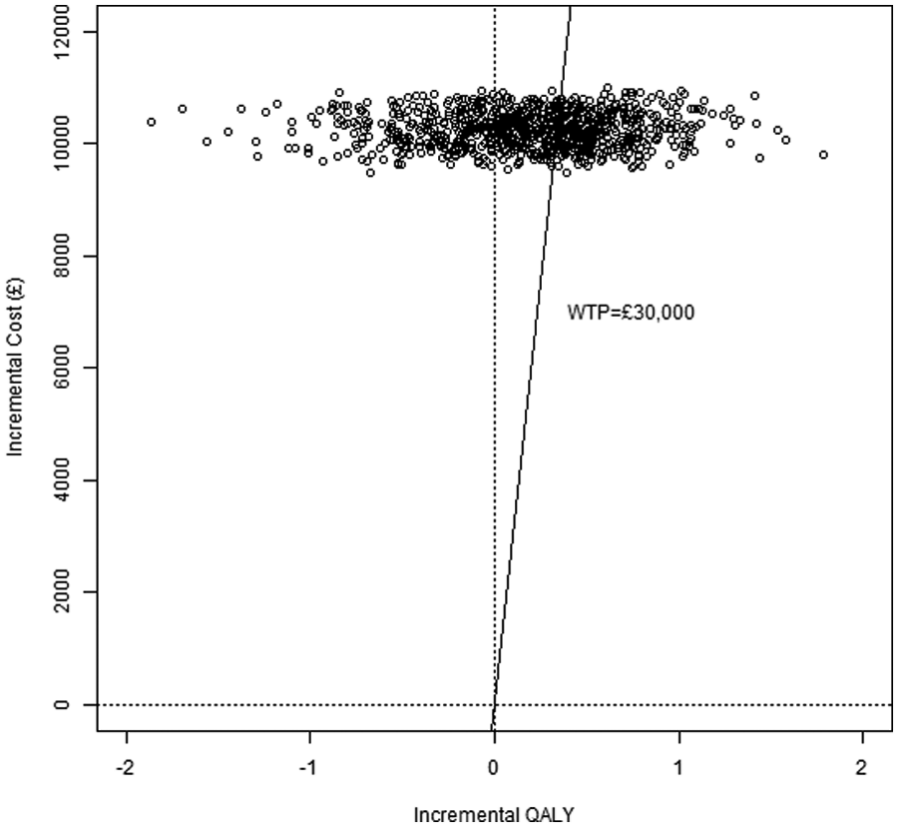
Cost-effectiveness plane. QALY, quality-adjusted life year; WTP, willingness to pay.

**Figure 8:**
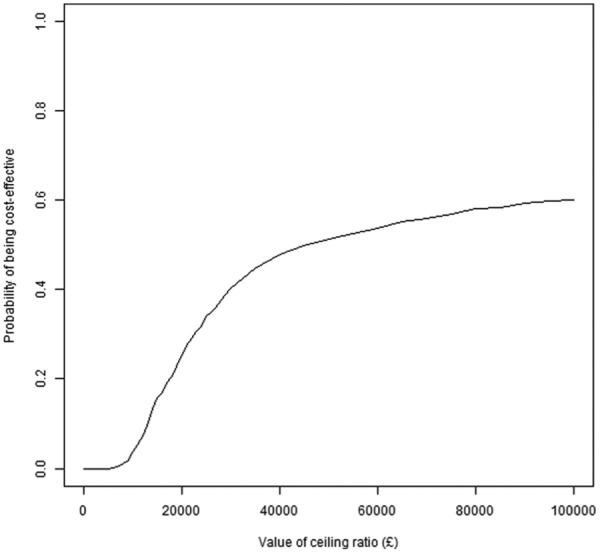
Cost-effectiveness acceptability curve.

Figure 7 shows the cost-effectiveness plane in this illustration. The probabilistic sensitivity analysis involved 1000 draws with 10% excluded due to computational difficulties related to differences in cumulative hazards between consecutive time points that were greater than 1. All draws resulted in the treatment of interest being more costly, and therefore the northwest and northeast quadrants are shown. The cost-effectiveness acceptability curve is shown in Figure 8. It can be seen in Figure 8 that, given a maximum willingness to pay of £100,000 per quality-adjusted life year gained, the probability that the treatment was cost-effective compared with the control was 0.60.

## Discussion

This article has introduced the state-arrival extended multi-state model and highlighted its potential as a tool to formally test the Markov property. We demonstrated its use in aiding the decision as to whether the Markovian assumption was reasonable, through the inclusion of a covariate representing patients’ history. In our illustration, the Markov property was not thought to be reasonable, and therefore the test result helped direct us to a semi-Markov, rather than Markov, approach to modeling.

The article has also provided a step-by-step guide to carrying out cost-effectiveness analysis in a multi-state modeling survival analysis framework and has provided R functions to build (state-arrival extended) Markov and semi-Markov models, calculate state occupancy probabilities and base-case mean life years/QALYs, and perform a full probabilistic sensitivity analysis. The particular illustration involved modeling each transition with treatment as the only covariate considered. This was because the individual patient-level data could not to be shared for the tutorial. However, the functions all have customizable arguments to allow the user to adapt the analysis to her or his data and requirements. For example, the model could include several covariates, have a different number of transitions, be based on different distributions for each transition, or have a different time horizon. When individual patient-level data are available from a trial or other study, we recommend making full use of the data. Incorporating all relevant predictors should be considered, provided the number of patients experiencing transitions was of sufficient size, and should lead to improved predictions of transition probabilities.

The existing functions in the mstate package in R can be adapted to accommodate any cumulative hazards chosen by the user. This tutorial has involved adapting Cox regression models to allow hazards to follow parametric distributions. A deterministic sensitivity analysis varying the treatment effect in the extrapolation period from that observed during the trial is also demonstrated. Piecewise modeling could be accommodated similarly by varying the hazards in different time periods within the observed and/or extrapolated section. Other approaches such as mixture modeling and splines could also be incorporated.

In using state-arrival extended models specifically to test the Markov property, we have used the effect of a covariate for time in the previous state to help decide whether the property held. A balance needs to be struck between the hypothesis testing results (assessment of a *P* value), which are influenced by sample size, and consideration of the practical importance of the effect size. With a large sample size, a statistically significant effect size could be too small to be of relevance, and therefore the analyst may wish to proceed as if the Markov property held. Conversely, with a small sample, there may not be evidence of statistical significance, but the effect size may be of practical importance, and therefore the analyst may decide that the Markov property is not reasonable.

Proportional hazard models have been involved in the modeling of transition hazards. Before considering such models, it is important to check that the PH assumption was reasonable for each of the covariates in the model. This illustration has involved the eyeballing of log-log and cumulative hazard v. time plots. We also recommend the use of the Grambsch-Therneau test for a more objective assessment of the PH assumption. If proportional hazards are not met, it is still possible to conduct an analysis. Options include fitting accelerated failure models or, with covariates that did not satisfy the PH requirement, fitting separate models for each level of a covariate, using time-dependent covariates or including an interaction with time.

When data at the individual patient level are not available, data can be obtained by digitizing published Kaplan-Meier curves, using, for example, the software Enguage,^20^ as in this illustration. Alternatively, Guyot and others^23^ have produced an algorithm that can be used to approximately reconstruct the data from a published Kaplan-Meier curve so that analysis can still take place. However, regardless of the method, there would need to be enough survival curves to represent all event times of interest. For instance, for the model illustrated in this article, a Kaplan-Meier estimate of overall survival, progression-free survival, *and* postprogression survival would be needed. With the data generated, Cox regression or parametric survival regression models could be fitted as if the actual patient data were available. Reconstructing the data from Kaplan-Meier survival curves can, however, limit the analysis to the “clock-reset” semi-Markov approach. When using the semi-Markov approach, the data used for the modeling of progression → death can be approximated from the postprogression Kaplan-Meier survival curve. However, this is not the case for the Markov approach. Because time is measured from the initial state in every transition in a Markov model, the modeling of progression → death requires knowledge—from the start of the study—of the time to progression and (possibly censored) time to death for each patient.

If individual patient-level data are not available, and survival curves are not comprehensive enough to approximate the data accurately enough, it may still be possible to undertake some analysis. If survival regression models are required, parameter estimates from previously published models can be used. If results of a semi-parametric Cox regression model are available, then cumulative hazards at each time point of interest can be derived and used with the built-in functions in mstate to create the state occupancy probabilities required. This, however, requires an estimate of the baseline hazard or survival. It also requires that the data used to create the model span the time horizon of interest. If parameter estimates from a parametric survival regression model are available, then these can be used as arguments in the functions with a noipd suffix on the dedicated Web page. It may be more appropriate to inform the inputs (i.e., cumulative hazards) needed for a transition from clinical information such as background mortality rate. The functions on the Web page with a noipd suffix also allow background mortality rates to be used for this purpose.

This particular illustration was used primarily to demonstrate an application of the method rather than to focus on the results of the cost-effectiveness analysis. In this example, clinical information was used to determine the time horizon. More generally a commonsense approach is needed to decide on the time horizon and the extrapolation to that point, particularly when there is uncertainty surrounding the cost-effectiveness in the unobserved period as in this case. External information such as registry data and/or expert opinion can be used to help provide extrapolation that is sensible.^24,25^

The state-arrival extended approach demonstrated in this article to test the Markov property required individual patient-level data in the sense that knowledge of (some function of) time in the previous state was necessary. If analysts wish to use a state-arrival extended approach, we recommend careful consideration of the covariate used to ensure it is clinically relevant.

In the probabilistic sensitivity analysis, we excluded 10% of the 1000 draws, due to computational difficulties related to differences in cumulative hazards between consecutive time points that were greater than 1. Therefore, bias may have been introduced. Including more time points at which to evaluate the transition probabilities would have rectified this. However, this was beyond the scope of this illustration. We believe the problem related to the cumulative hazards was primarily due to the shape and scale parameters in the Gompertz distributions used and that other distributions would not exhibit the problem to the same extent.

Although health economic modeling in spreadsheet packages has its advantages, we consider this multi-state modeling approach an attractive alternative worth considering. We hope this article, and the accompanying functions, will encourage health economists to use this approach.

## Supplementary Material

Supplementary material

Supplementary material
