# Supplementary material for: Cost-effectiveness Analysis in R Using a Multi-state Modeling Survival Analysis Framework: A Tutorial
Source: Med Decis Making. 2016 Jun 8;37(4):340–52. doi: 10.1177/0272989X16651869 (PMC5424858; doi:10.1177/0272989X16651869)
Supplement: Supplementary material [file Appendix1.pdf]

Appendix 1

Choice of distribution for each transition

progression -> death

Progression -> death was considered in the main article with the Gompertz distribution chosen for that transition.

progression –free -> progression

For brevity, only the RFC treatment arm is shown.

Figure A1 shows - over the trial observation period - the observed proportion of being in the progression state, alongside predicted probabilities from the semi-Markov models. Each colour represents one of six different distributions used to fit progression-free -> progression. The different shades of a particular colour represent the six different distributions used to fit progression-free -> death. The predictions of different shades of the same colour were similar indicating that the predictions of progression-free -> progression were not sensitive to the distribution used for progression-free -> death. Progression -> death was fitted using a Gompertz distribution.

Figure A1 Probability of being in the progression state: trial observation period

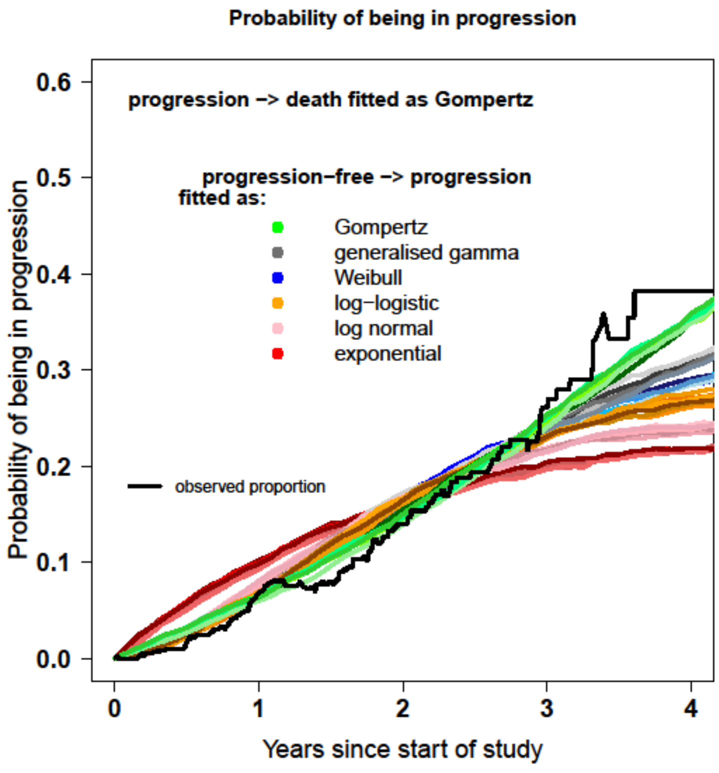

It can be seen from Figure A1 that the Gompertz distribution appeared to provide the most reasonable fit.

Figure A2 shows the predicted probabilities of being in the progression state from the semi-Markov models extrapolated to 15 years.

Figure A2 Probability of being in the progression state: extrapolation to 15 years

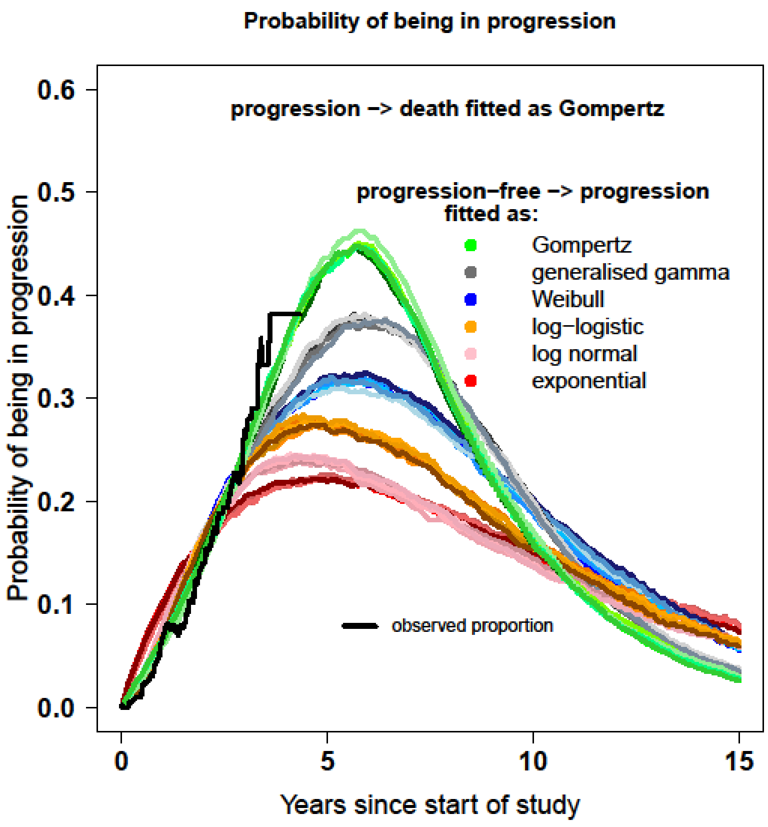

Figure A2 suggests the Gompertz or generalised gamma distributions provide the most sensible extrapolation as they are closest to reaching zero by 15 years. The Gompertz distribution had the highest peak and was closest to the observed data for longer than the other distributions.

A Gompertz distribution was chosen for the progression-free -> progression transition.

progression-free -> death

Figure A3 shows - over the trial observation period - the observed proportion in progression-free -> death, alongside predicted probabilities from the semi-Markov models. Progression -> death and progression-free -> progression were fitted using Gompertz distributions.   Only the RFC treatment arm is shown.

Figure A3 Progression-free -> death: trial observation period

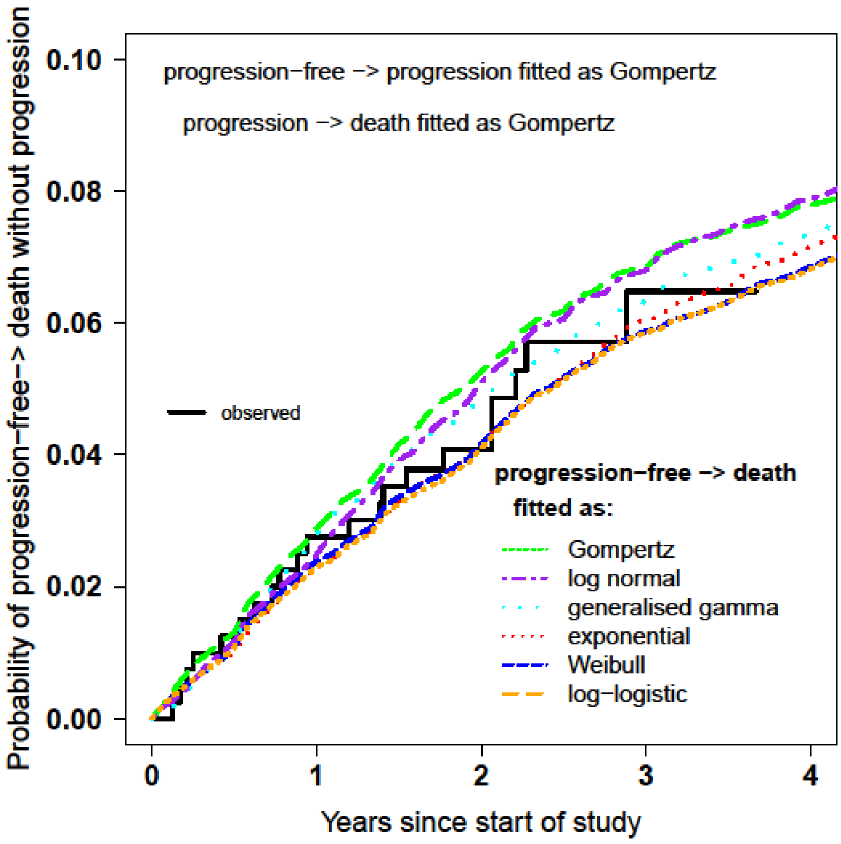

The distributions produced very similar fits over the first year before diverging. The generalised gamma, log normal and Gompertz distributions appeared to provide the most reasonable predictions.

Figure A4 shows the predicted probabilities of being in progression-free -> death from the semi-Markov models extrapolated to 15 years.

Figure A4 Progression-free -> death: extrapolation to 15 years

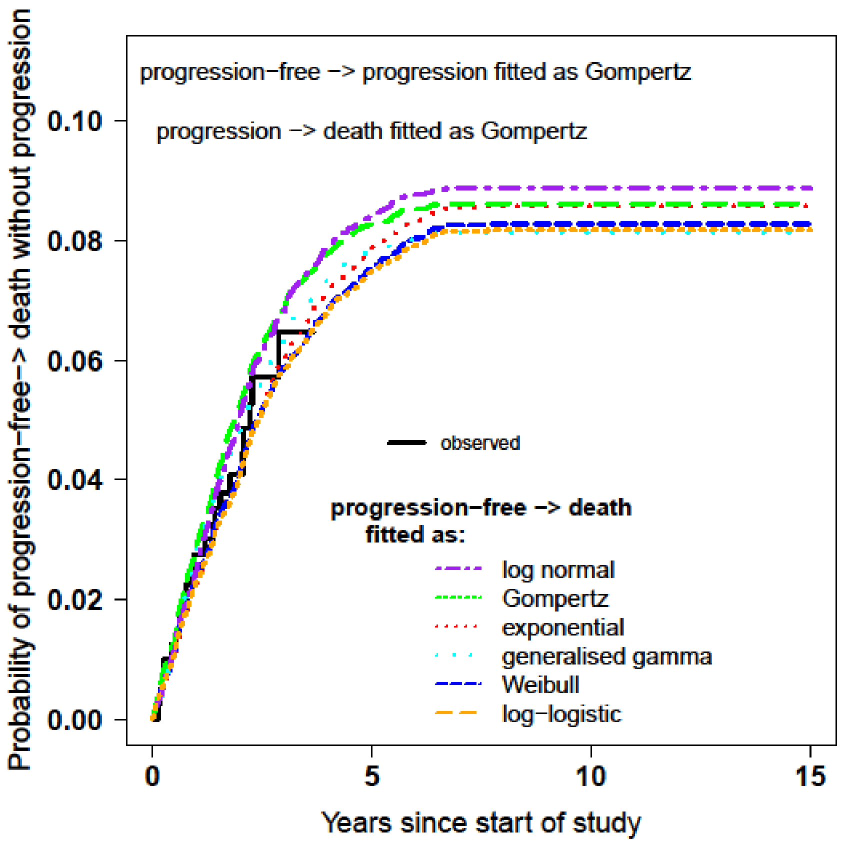

There was very little to choose between the distributions. A generalised gamma distribution was chosen for the progression-free -> death transition because it appeared to achieve the best balance of a reasonable fit to the observed data and a sensible extrapolation. A sensitivity analysis considering alternative fits for each transition is considered in Appendix 2. The models used for the base case for each transition are shown below.

ONLINE SUPPLEMENTARY MATERIAL

BASE CASE MODEL

Progression-free -> progression (Gompertz)

Covariate	Coefficient	se (Coefficient)	p-value
treatment	-0.549	0.129	<0.001
gamma	0.510	0.071	<0.001
constant	-2.280	0.134	<0.001

Progression-free -> death (generalised gamma)

Covariate	Coefficient	se (Coefficient)	p-value
mu	4.256	0.536	<0.001
sigma	3.316	1.953	
kappa	-0.721	1.556	0.643
treat	0.461	0.345	0.182
log(sigma)	1.199	0.589	0.042

Progression -> death (Gompertz)

Covariate	Coefficient	se (Coefficient)	p-value
treatment	0.229	0.300	0.445
gamma	0.037	0.238	0.877
constant	-1.576	0.266	<0.001
