# Supplementary material for: Cost-effectiveness Analysis in R Using a Multi-state Modeling Survival Analysis Framework: A Tutorial
Source: Med Decis Making. 2016 Jun 8;37(4):340–52. doi: 10.1177/0272989X16651869 (PMC5424858; doi:10.1177/0272989X16651869)
Supplement: Supplementary material [file Appendix2.pdf]

Appendix 2 Sensitivity analysis considering alternative distributions for each transition

The following table shows the Cost per QALYs gained when each of the six distributions were used for each transition. The vast majority of the Cost per QALY gained were below £30,000. However each time a Gompertz distribution was used for the progression-free -> progression transition the Cost per QALY gained exceeded £30,000.

Distribution for:					
progression-free -> progression	progression-free -> death	progression -> death	incremental QALYs	incremental Costs	Cost per QALY gained
**Gompertz**	**generalised gamma**	**Gompertz**	**0.276**	**£10,408**	**£37,665**
exponential	Gompertz	Weibull	0.841	£9,803	£11,657
exponential	Gompertz	Gompertz	0.812	£9,725	£11,978
exponential	Weibull	Weibull	0.819	£9,836	£12,009
exponential	Gompertz	exponential	0.794	£9,617	£12,105
exponential	generalised gamma	Gompertz	0.791	£9,688	£12,252
exponential	generalised gamma	exponential	0.778	£9,587	£12,317
exponential	generalised gamma	Weibull	0.785	£9,676	£12,323
exponential	log normal	Weibull	0.782	£9,700	£12,401
exponential	exponential	Weibull	0.793	£9,860	£12,437
exponential	Gompertz	log-logistic	0.784	£9,761	£12,452
exponential	log-logistic	Weibull	0.791	£9,865	£12,464
exponential	Weibull	Gompertz	0.769	£9,701	£12,612
exponential	log normal	Gompertz	0.761	£9,603	£12,626
exponential	Gompertz	generalised gamma	0.748	£9,484	£12,673
exponential	generalised gamma	log-logistic	0.762	£9,661	£12,686
exponential	Weibull	exponential	0.754	£9,571	£12,698
exponential	exponential	Gompertz	0.762	£9,729	£12,767
exponential	log-logistic	Gompertz	0.758	£9,748	£12,866
exponential	log normal	exponential	0.740	£9,537	£12,883
exponential	exponential	exponential	0.741	£9,671	£13,058
exponential	Gompertz	log normal	0.736	£9,629	£13,090
exponential	generalised gamma	generalised gamma	0.716	£9,386	£13,112
exponential	Weibull	log-logistic	0.742	£9,825	£13,237
exponential	log normal	log-logistic	0.729	£9,656	£13,244
exponential	log-logistic	exponential	0.723	£9,598	£13,272
exponential	log normal	generalised gamma	0.703	£9,370	£13,331
exponential	generalised gamma	log normal	0.712	£9,526	£13,375
exponential	exponential	log-logistic	0.740	£9,903	£13,386
exponential	Weibull	generalised gamma	0.709	£9,632	£13,589
exponential	log normal	log normal	0.696	£9,514	£13,668
exponential	exponential	generalised gamma	0.708	£9,697	£13,690
exponential	log-logistic	log-logistic	0.698	£9,710	£13,913
exponential	log-logistic	log normal	0.687	£9,634	£14,019
exponential	log-logistic	generalised gamma	0.677	£9,509	£14,036
exponential	Weibull	log normal	0.682	£9,646	£14,139

exponential	exponential	log normal	0.658	£9,656	£14,672
log normal	Weibull	Weibull	0.686	£10,139	£14,773
log normal	Weibull	log-logistic	0.684	£10,124	£14,794
log normal	log normal	Weibull	0.660	£9,856	£14,937
log normal	exponential	log-logistic	0.664	£10,013	£15,069
log normal	log-logistic	Weibull	0.674	£10,179	£15,094
log normal	log-logistic	log-logistic	0.666	£10,066	£15,103
log normal	log-logistic	Gompertz	0.666	£10,096	£15,153
log normal	exponential	Weibull	0.658	£10,004	£15,204
log normal	Weibull	Gompertz	0.663	£10,084	£15,214
log-logistic	Gompertz	Gompertz	0.663	£10,214	£15,398
log normal	log-logistic	log normal	0.646	£9,958	£15,412
log normal	Weibull	log normal	0.642	£10,000	£15,574
log normal	Weibull	generalised gamma	0.636	£9,921	£15,589
log normal	log-logistic	generalised gamma	0.635	£9,903	£15,605
log normal	Weibull	exponential	0.644	£10,056	£15,620
log normal	exponential	generalised gamma	0.624	£9,789	£15,677
log normal	Gompertz	Weibull	0.633	£9,944	£15,698
log normal	exponential	Gompertz	0.635	£9,965	£15,699
log normal	generalised gamma	Weibull	0.622	£9,775	£15,720
log normal	log normal	Gompertz	0.621	£9,795	£15,762
log normal	Gompertz	log-logistic	0.637	£10,152	£15,935
log normal	Gompertz	Gompertz	0.612	£9,873	£16,124
log normal	log-logistic	exponential	0.614	£9,993	£16,265
log normal	exponential	exponential	0.608	£9,887	£16,270
log-logistic	Gompertz	exponential	0.622	£10,123	£16,281
log normal	generalised gamma	log-logistic	0.609	£9,915	£16,291
log normal	log normal	log-logistic	0.616	£10,038	£16,292
log-logistic	Gompertz	Weibull	0.622	£10,141	£16,311
log normal	log normal	exponential	0.596	£9,818	£16,470
log-logistic	exponential	log-logistic	0.621	£10,224	£16,471
log normal	exponential	log normal	0.597	£9,837	£16,480
log-logistic	log-logistic	exponential	0.609	£10,077	£16,559
log-logistic	log-logistic	log-logistic	0.614	£10,233	£16,675
log-logistic	log-logistic	Weibull	0.606	£10,116	£16,694
log normal	Gompertz	log normal	0.601	£10,037	£16,709
log normal	generalised gamma	Gompertz	0.581	£9,713	£16,731
log normal	Gompertz	generalised gamma	0.588	£9,852	£16,748
log-logistic	Gompertz	log-logistic	0.614	£10,293	£16,764
log-logistic	Weibull	log-logistic	0.603	£10,231	£16,955
log-logistic	log-logistic	Gompertz	0.599	£10,181	£16,987
log-logistic	log normal	log-logistic	0.610	£10,363	£16,989
log normal	generalised gamma	generalised gamma	0.552	£9,522	£17,236
log normal	generalised gamma	exponential	0.557	£9,615	£17,267
log normal	log normal	log normal	0.570	£9,844	£17,277
log-logistic	exponential	Weibull	0.591	£10,218	£17,295
log-logistic	exponential	exponential	0.581	£10,084	£17,345
log-logistic	Weibull	Weibull	0.586	£10,210	£17,418
log normal	Gompertz	exponential	0.567	£9,898	£17,471
log-logistic	log-logistic	log normal	0.577	£10,083	£17,484
log normal	log normal	generalised gamma	0.554	£9,712	£17,521
log-logistic	Weibull	exponential	0.581	£10,206	£17,572

log-logistic	log normal	Weibull	0.577	£10,142	£17,590
log-logistic	exponential	generalised gamma	0.567	£9,973	£17,596
Weibull	log normal	Weibull	0.588	£10,368	£17,645
log normal	generalised gamma	log normal	0.556	£9,810	£17,648
Weibull	log-logistic	exponential	0.584	£10,324	£17,687
log-logistic	exponential	Gompertz	0.573	£10,173	£17,763
log-logistic	exponential	log normal	0.561	£9,966	£17,769
Weibull	Weibull	Weibull	0.573	£10,195	£17,798
log-logistic	log normal	exponential	0.564	£10,072	£17,860
log-logistic	generalised gamma	Weibull	0.569	£10,215	£17,941
log-logistic	Weibull	Gompertz	0.571	£10,266	£17,970
log-logistic	generalised gamma	Gompertz	0.561	£10,108	£18,009
log-logistic	log-logistic	generalised gamma	0.552	£9,966	£18,048
log-logistic	generalised gamma	log-logistic	0.568	£10,278	£18,096
Weibull	log normal	Gompertz	0.570	£10,368	£18,175
log-logistic	Weibull	generalised gamma	0.548	£10,043	£18,322
log-logistic	Gompertz	generalised gamma	0.548	£10,059	£18,371
log-logistic	Weibull	log normal	0.544	£10,035	£18,446
log-logistic	log normal	Gompertz	0.544	£10,086	£18,544
Weibull	log-logistic	log normal	0.560	£10,383	£18,555
log-logistic	Gompertz	log normal	0.543	£10,108	£18,599
log-logistic	log normal	generalised gamma	0.540	£10,089	£18,686
Weibull	Gompertz	Weibull	0.539	£10,196	£18,904
Weibull	log-logistic	log-logistic	0.548	£10,380	£18,934
Weibull	log normal	generalised gamma	0.540	£10,218	£18,936
Weibull	log-logistic	Weibull	0.543	£10,328	£19,028
Weibull	generalised gamma	log-logistic	0.544	£10,359	£19,033
Weibull	Weibull	log-logistic	0.537	£10,240	£19,060
Weibull	log normal	log-logistic	0.549	£10,484	£19,108
log-logistic	log normal	log normal	0.528	£10,093	£19,117
Weibull	exponential	log-logistic	0.533	£10,207	£19,138
log-logistic	generalised gamma	exponential	0.516	£9,937	£19,272
Weibull	log normal	exponential	0.524	£10,139	£19,345
Weibull	exponential	exponential	0.511	£10,093	£19,737
log-logistic	generalised gamma	generalised gamma	0.503	£9,940	£19,748
log-logistic	generalised gamma	log normal	0.517	£10,223	£19,787
Weibull	Weibull	exponential	0.512	£10,140	£19,793
Weibull	Gompertz	Gompertz	0.508	£10,100	£19,870
Weibull	log-logistic	Gompertz	0.506	£10,098	£19,957
Weibull	Gompertz	exponential	0.505	£10,109	£20,020
Weibull	Gompertz	log-logistic	0.521	£10,454	£20,062
Weibull	Weibull	generalised gamma	0.511	£10,254	£20,062
Weibull	generalised gamma	Weibull	0.501	£10,064	£20,075
Weibull	exponential	Weibull	0.502	£10,098	£20,125
Weibull	Weibull	log normal	0.508	£10,264	£20,208
Weibull	exponential	generalised gamma	0.504	£10,257	£20,342
Weibull	generalised gamma	exponential	0.500	£10,184	£20,387
Weibull	log-logistic	generalised gamma	0.507	£10,388	£20,490
Weibull	generalised gamma	generalised gamma	0.498	£10,216	£20,529
Weibull	Weibull	Gompertz	0.482	£10,063	£20,892
Weibull	Gompertz	generalised gamma	0.481	£10,190	£21,179
Weibull	exponential	log normal	0.483	£10,253	£21,227

Weibull	exponential	Gompertz	0.473	£10,104	£21,348
generalised gamma	Weibull	log-logistic	0.497	£10,629	£21,377
generalised gamma	log-logistic	log-logistic	0.481	£10,594	£22,038
generalised gamma	exponential	log-logistic	0.481	£10,699	£22,261
Weibull	log normal	log normal	0.466	£10,378	£22,268
Weibull	generalised gamma	Gompertz	0.435	£9,980	£22,920
Weibull	Gompertz	log normal	0.446	£10,258	£22,980
generalised gamma	log-logistic	Gompertz	0.449	£10,453	£23,296
Weibull	generalised gamma	log normal	0.431	£10,083	£23,375
generalised gamma	log-logistic	generalised gamma	0.442	£10,451	£23,658
generalised gamma	Weibull	Gompertz	0.446	£10,546	£23,673
generalised gamma	Weibull	generalised gamma	0.436	£10,477	£24,025
generalised gamma	exponential	Gompertz	0.433	£10,504	£24,282
generalised gamma	Gompertz	log-logistic	0.434	£10,614	£24,479
generalised gamma	Gompertz	Gompertz	0.422	£10,556	£24,994
generalised gamma	Gompertz	generalised gamma	0.420	£10,554	£25,098
generalised gamma	Gompertz	Weibull	0.404	£10,263	£25,395
generalised gamma	Weibull	Weibull	0.404	£10,288	£25,459
generalised gamma	exponential	log normal	0.414	£10,577	£25,571
generalised gamma	generalised gamma	Weibull	0.398	£10,227	£25,667
generalised gamma	exponential	generalised gamma	0.401	£10,351	£25,796
generalised gamma	generalised gamma	log-logistic	0.404	£10,445	£25,863
generalised gamma	Weibull	log normal	0.407	£10,579	£26,007
generalised gamma	log-logistic	Weibull	0.389	£10,242	£26,295
generalised gamma	log normal	Gompertz	0.401	£10,557	£26,328
generalised gamma	log-logistic	exponential	0.394	£10,433	£26,455
generalised gamma	Weibull	exponential	0.392	£10,399	£26,514
generalised gamma	generalised gamma	exponential	0.387	£10,281	£26,588
generalised gamma	log normal	Weibull	0.385	£10,254	£26,625
generalised gamma	generalised gamma	generalised gamma	0.389	£10,395	£26,707
generalised gamma	log normal	log-logistic	0.389	£10,402	£26,731
generalised gamma	exponential	Weibull	0.373	£10,151	£27,205
generalised gamma	generalised gamma	Gompertz	0.374	£10,442	£27,898
generalised gamma	Gompertz	log normal	0.374	£10,495	£28,042
generalised gamma	log-logistic	log normal	0.372	£10,487	£28,154
generalised gamma	log normal	generalised gamma	0.365	£10,288	£28,177
generalised gamma	log normal	exponential	0.343	£10,130	£29,547
generalised gamma	exponential	exponential	0.346	£10,216	£29,555
generalised gamma	Gompertz	exponential	0.340	£10,253	£30,114
generalised gamma	generalised gamma	log normal	0.335	£10,284	£30,727
Gompertz	generalised gamma	Weibull	0.299	£10,498	£35,101
Gompertz	generalised gamma	log-logistic	0.301	£10,654	£35,378
generalised gamma	log normal	log normal	0.287	£10,277	£35,855
Gompertz	exponential	Weibull	0.288	£10,494	£36,387
Gompertz	exponential	log-logistic	0.279	£10,652	£38,180
Gompertz	Gompertz	log-logistic	0.277	£10,596	£38,311
Gompertz	Weibull	Weibull	0.268	£10,489	£39,107
Gompertz	Gompertz	Weibull	0.267	£10,499	£39,275
Gompertz	log normal	Weibull	0.263	£10,453	£39,675
Gompertz	log-logistic	Weibull	0.261	£10,484	£40,124
Gompertz	Weibull	log-logistic	0.258	£10,535	£40,825
Gompertz	generalised gamma	generalised gamma	0.256	£10,469	£40,928

Gompertz	log-logistic	log-logistic	0.254	£10,514	£41,368
Gompertz	log normal	log-logistic	0.255	£10,608	£41,545
Gompertz	Weibull	Gompertz	0.239	£10,346	£43,255
Gompertz	exponential	Gompertz	0.231	£10,289	£44,564
Gompertz	log-logistic	Gompertz	0.228	£10,289	£45,046
Gompertz	generalised gamma	exponential	0.226	£10,367	£45,881
Gompertz	Gompertz	exponential	0.217	£10,308	£47,489
Gompertz	exponential	generalised gamma	0.218	£10,350	£47,510
Gompertz	Weibull	log normal	0.218	£10,364	£47,547
Gompertz	Gompertz	generalised gamma	0.215	£10,324	£48,088
Gompertz	log-logistic	log normal	0.211	£10,358	£49,016
Gompertz	Gompertz	Gompertz	0.208	£10,313	£49,560
Gompertz	exponential	exponential	0.205	£10,347	£50,529
Gompertz	Weibull	generalised gamma	0.204	£10,332	£50,756
Gompertz	exponential	log normal	0.203	£10,361	£51,107
Gompertz	Weibull	exponential	0.201	£10,290	£51,176
Gompertz	log-logistic	generalised gamma	0.193	£10,305	£53,484
Gompertz	log normal	Gompertz	0.189	£10,282	£54,389
Gompertz	Gompertz	log normal	0.190	£10,394	£54,843
Gompertz	log-logistic	exponential	0.179	£10,243	£57,228
Gompertz	log normal	generalised gamma	0.159	£10,277	£64,661
Gompertz	log normal	exponential	0.150	£10,233	£68,307
Gompertz	generalised gamma	log normal	0.142	£10,173	£71,859
Gompertz	log normal	log normal	0.140	£10,347	£73,914
